# Care trajectories in children with profound intellectual and multiple disabilities/polyhandicap: a cross-sectional study of the French National Cohort

**DOI:** 10.3389/fpubh.2026.1873749

**Published:** 2026-07-03

**Authors:** Karine Baumstarck, Souad Loukkal, Houria El Ouazzani, Sibylle Del Duca, Any Beltran, Ilyes Hamouda, Marie-Christine Rousseau

**Affiliations:** 1 EA 3279, CEReSS - Research Centre on Health Services and Quality of Life, Aix Marseille University, Marseille, France; 2 Epidemiology and Health Economy Department, Aix Marseille University, Marseille, France; 3 Fédération des Hôpitaux de Polyhandicap et Multihandicap, San Salvadour Hospital, University Hospital of Paris, Hyères, France

**Keywords:** care management, care trajectories, children, PIMD, polyhandicap

## Abstract

**Background:**

There are few studies that objectively describe the care trajectories of individuals with profound intellectual and multiple disabilities (PIMD/polyhandicap). This study aims to provide an initial description of these care trajectories from birth to 18 years.

**Methods:**

The cross-sectional study used data from the French cohort (EVALuation PoLyHandicap, EVAL-PLH) of individuals with PIMD/polyhandicap. Data were collected between 2020 and 2021. Inclusion criteria were: (i) age older than 3 years at the time of inclusion; (ii) a diagnosis of polyhandicap defined as an early brain lesion causing a combination of severe motor impairment, profound intellectual disability, and high dependency in daily life. To ensure data reliability, the analysis focused on individuals under 35 years of age. Four life periods were considered: 0–2 years, 3–5 years, 6–10 years, and 11–17 years. For each period, the primary care modality—defined as the care setting in which the individual spent the majority of time—was recorded (nursery, exclusive home care, day care facility, 24-h care facility). Care trajectories were analyzed using Sankey diagrams.

**Results:**

A total of 407 individuals were included in the analysis. During the 0–2-year period, a large proportion of the sample was cared for exclusively at home, but this proportion decreased with age. In contrast, the use of 24-h care facilities showed an increasing trend over time and became the predominant care modality during the 11–17-year period. Day care facilities never represented the dominant care modality in any life period. The time spent in the primary care modality ranged from 22 to 47 months across the four periods. Less than 5% of individuals experienced a care discontinuity event.

**Conclusion:**

This study provides initial empirical evidence describing the care trajectories of individuals with PIMD/polyhandicap from birth to 18 years. These findings offer a valuable basis for evaluating current service provision and identifying gaps in the organization of care. A better understanding of these trajectories may help inform policy decisions, improve the quality and accessibility of services, and support more appropriate allocation of resources for this population.

**Clinical trial registration:**

Clinical trial registration number NCT02400528 registered 27/03/2015.

## 1 Introduction

Profound intellectual and multiple disability (PIMD) or polyhandicap (1) refers to a severe disability resulting from early damage to an immature brain, usually before the age of 3. It is characterized by a combination of profound intellectual disability and severe motor impairment. When the disorder affects an immature brain, the term of polyhandicap is used; polyhandicap, as a subgroup of PIMD, includes the most severe cases due to the early onset of the brain damage. Recently, the Ithaca European Reference Network for congenital malformations and rare intellectual disabilities has adopted the combined term PIMD/polyhandicap (1). The resulting condition leads to profound dependency, requiring lifelong, continuous support across all domains of daily life, including medical care, mobility, communication, and basic activities. This support is provided either by family caregivers, primarily the parents, or by institutional caregivers working in specialized facilities dedicated to the care of these individuals.

Care responsibilities are shared between family caregivers and institutional caregivers, and vary depending on care modalities and the organization of the healthcare system (2). Some countries promote deinstitutionalization by enabling families to keep the person at home, identifying their needs, and providing the necessary human and financial support (3, 4). In other countries, such as France for example, care in institutions is more common and widely used (5). In France, care for individuals with PIMD/polyhandicap is largely publicly funded and organized across both the healthcare and medico-social sectors, shaping resource allocation, service provision, and the respective roles of families and institutions (2). In this context, care is generally divided into three main categories. First, home-based care, which mostly concerns very young children, corresponds to individuals exclusively cared for at home, with no contact with specific care facilities. Then, institutional care may be delivered either on a part-time basis—such as day care only—or on a full-time basis, where individuals stay both day and night. Among the full-time care options, the ‘nursery’ represents a specific type of institution dedicated to caring for young children (generally aged 0 to 3). Some of these facilities are specialized in providing medical care, and in certain cases, children with PIMD/polyhandicap are also placed in these settings. However, there is a lack of robust data quantifying the distribution between these different forms of care (6).

To our knowledge, there are very few studies, nationally or internationally, that clearly provide an objective description of the care trajectories of these individuals (7). Available information mainly comes from families and healthcare professionals, which highlight the major difficulties and misunderstandings experienced by parents throughout the care pathway. Several barriers limit optimal care management for these individuals, including lack of knowledge about available care facilities and their locations, limited awareness of home-based medical and paramedical support services, and long delays for admission into specialized institutions (8, 9). Beyond these qualitative reports, little is known about how care is actually distributed over time in this population, including the proportion of families who keep their child at home and for how long, as well as the timing and reasons underlying placement or non-placement in institutional settings. A better understanding of these elements could help clarify the factors that drive transitions between different modalities of care (home care, nursery care, full-time residential care, or day-care facilities). In particular, documenting these trajectories may help to better understand the difficulties reported by families, including challenges related to service availability, accessibility, coordination of care, and financial or organizational constraints. Moreover, the respective influence of clinical, developmental, family and economic factors on these decisions and trajectories remains poorly understood.

Improving knowledge of these care trajectories may provide valuable insights for policymakers, enabling them to identify shortcomings and unmet needs, and to better target policies aimed at enhancing the quality and accessibility of care in this under-recognized area (10–12). Our central research question is: in this population, where do critical transitions or discontinuities in care tend to arise? This study, based on a large sample from the French national cohort EVAL-PLH (EVALuation PoLyHandicap), aims for the first time to provide an objective description of these care trajectories.

## 2 Methods

### 2.1 Design and settings

The study used data from the French cohort (EVALuation PoLyHandicap EVAL-PLH) of individuals with PIMD/polyhandicap. The general aim of the cohort is to study the impact of socioeconomic, environmental, and epidemiologic determinants on the health status of individuals and the daily life of their (institutional and familial) caregivers. Details of the study protocol of the cohort were published elsewhere (13). Data were collected at 3 points: 2015–2016, 2020–2021, and 2025–2026. The cohort includes 12 participating centers, comprising both day care facilities and 24-h care facilities.

### 2.2 Selection criteria

The inclusion criteria for the persons with PIMD/polyhandicap were as follows: age over 3 years at the time of inclusion; polyhandicap defined by: (i) a cerebral lesion leading to a combination of motor deficiency (tetraparesia, hemiparesis, paraparesis, extrapyramidal syndrome, cerebellar syndrome, and/or neuromuscular problems), profound intellectual impairment (intelligence quotient IQ < 40) associated with everyday life dependence (FIM < 55), and restricted mobility (GMFCS III, IV, and V); (ii) age at onset of the cerebral lesion younger than 3 years old; and (iii) usual care setting in specialized centres (including day care facilities and 24-h care facilities).

Because information on care trajectories for older individuals was considered less reliable and more inconsistently collected, the sample was restricted to individuals under 35 years of age at the time of assessment to ensure data robustness.

### 2.3 Data collection

This study examines data collected during the second point, in 2020–2021. The data included the following: sex, age, lesion time (antenatal, perinatal, and postnatal), aetiology nature (non-progressive and progressive), severity of health status (a severe case is defined by the combination of severe motor handicap (including paraparesis, tetraparesia, extrapyramidal syndrome, or severe general hypotonia), intellectual quotient IQ < 25, severe dependence defined by a FIM score ≤20, and severe motor deficit defined by a Gross Motor Function Classification System GMFCS IV-V), stability of health status [an unstable case is defined by the presence of at least one of the following criteria: recurrent pulmonary infections (≥5/year) and-or drug-resistant epilepsy (≥4 seizures/month)], mobility (GMFCS), independency (Functional Independency Measure FIM), number of medical devices (invasive mechanical ventilation, noninvasive mechanical ventilation, tracheostomy, nasogastric tube, gastrostomy, permanent urinary probe, cerebrospinal fluid derivation, and central venous catheter).

### 2.4 Care trajectories

For each person, data collection was divided into four life periods: from birth to 2 years of age, from 3 to 5 years of age, from 6 to 10 years of age, and from 11 to 17 years of age. For each life period, the primary care modality (defined as the one where the individual spent the majority of time in the period) was recorded, including nursery, exclusively home care, day care facility, and 24-h care facility. The following definitions were used: (1) Nursery: individuals cared for in this specific type of childcare institution; (2) Exclusively home care: individuals cared for at home, with no involvement with specific care facilities; (3) Day care facility: individuals cared for and supervised during the day but returning home in the evening; (4) 24-h care facility: individuals primarily cared for both during the day and at night.

The mean duration (in months) of time spent in the primary care modality, as well as the proportion of time spent in the primary care modality, were collected for each period.

For each period, care discontinuity events were collected. Care discontinuity is defined as an interruption in the continuity of care provision, occurring when support or services from a care facility or structure are unexpectedly disrupted. Planned transitions or temporary hospitalizations were not considered as care discontinuity events.

### 2.5 Ethics statement

Regulatory monitoring was performed in accordance with French law that requires the approval of the French ethics committee (Comité de Protection des Personnes Sud Méditerranée V, 20/10/2014, reference number 2014-A00953-44). A written consent form was collected for each participant (from the legal representative). Clinical trial number: NCT02400528 (registered 27/03/2015).

### 2.6 Statistical analysis

Descriptive data of the sample was provided. The care trajectories were analyzed using the Sankey diagrams. Origins, targets, and flow values of care modalities over different life periods were used. The diagrams illustrate the transitions among the four care modalities across the four predefined life periods ([0–2], [3–5], [6–10], and [11–17] years of age). The care modalities were classified as nursery, home, day care facility, and 24-h care facility. The diagram was provided for the full sample and for a subsample of individuals aged 18 to 35 years, representing individuals whose age is beyond the last predefined life period.

The mean durations, the proportions of time spent in the primary care modality, and the care discontinuity events were illustrated using histograms.

## 3 Results

### 3.1 Sample

Of the 623 individuals included in the second wave of the EVAL-PLH cohort, 407 were under 35 years of age and retained for analysis. The main characteristics of the sample is provided in Table 1. The sex ratio (male-to-female) was 1.18. At the time of assessment, 51% (*N* = 205) of the sample were over 18 years of age. Approximately 75% (*N* = 310) of the participants received care in 24-h care facilities (*N* = 95), whereas 25% attended day care facilities.

**Table 1:** Sample characteristics.

Characteristic	Category	Value	*N* = 407
Sex	Boys/men	*N* (%)	220 (54.1)
	Girls/women	*N* (%)	187 (45.9)
Age (years)		*M* (SD)	18.8 (7.6)
Age classes	3–17 years	*N* (%)	202 (49.6)
	18–35 years	*N* (%)	205 (50.4)
Lesion time	Antenatal	*N* (%)	200 (56.7)
	Perinatal	*N* (%)	75 (21.2)
	Postnatal	*N* (%)	55 (15.6)
	Non-classifiable	*N* (%)	23 (6.5)
Aetiology nature	Nonprogressive	*N* (%)	240 (68.0)
	Progressive	*N* (%)	89 (25.2)
	Nonclassifiable	*N* (%)	24 (6.8)
Health severity	Less severe	*N* (%)	205 (53.2)
	Severe	*N* (%)	180 (46.8)
Health stability	Stable	*N* (%)	290 (79.2)
	Unstable	*N* (%)	76 (20.8)
Mobility (GMFCS)	III	*N* (%)	53 (13.1)
	IV	*N* (%)	24 (5.9)
	V	*N* (%)	327 (80.9)
Profound intellectual impairment	Yes	*N* (%)	382 (87.4)
	No	*N* (%)	10 (2.6)
At least 1 medical device	Yes	*N* (%)	200 (50.4)
	No	*N* (%)	197 (49.6)

*N* (%): number (percent); *M* (SD): mean (standard deviation). Health severity: a severe case is defined by the combination of motor handicap (including paraparesis, tetraparesia, extrapyramidal syndrome, or severe general hypotonia), intellectual quotient IQ <25, severe dependence defined by a FIM score ≤20, and severe motor deficit defined by a GMFCS IV-V. Health stability: recurrent pulmonary infections (≥5/year) and-or drug-resistant epilepsy (≥4 seizures/month). GMFCS: Gross Motor Function Scale. Profound intellectual impairment: Intelligence quotient <25 or non-evaluable. Medical devices: invasive mechanical ventilation, noninvasive mechanical ventilation, tracheostomy, nasogastric tube, gastrostomy, permanent urinary probe, cerebrospinal fluid derivation, and central venous catheter.

### 3.2 Care trajectories

**Figure 1:**
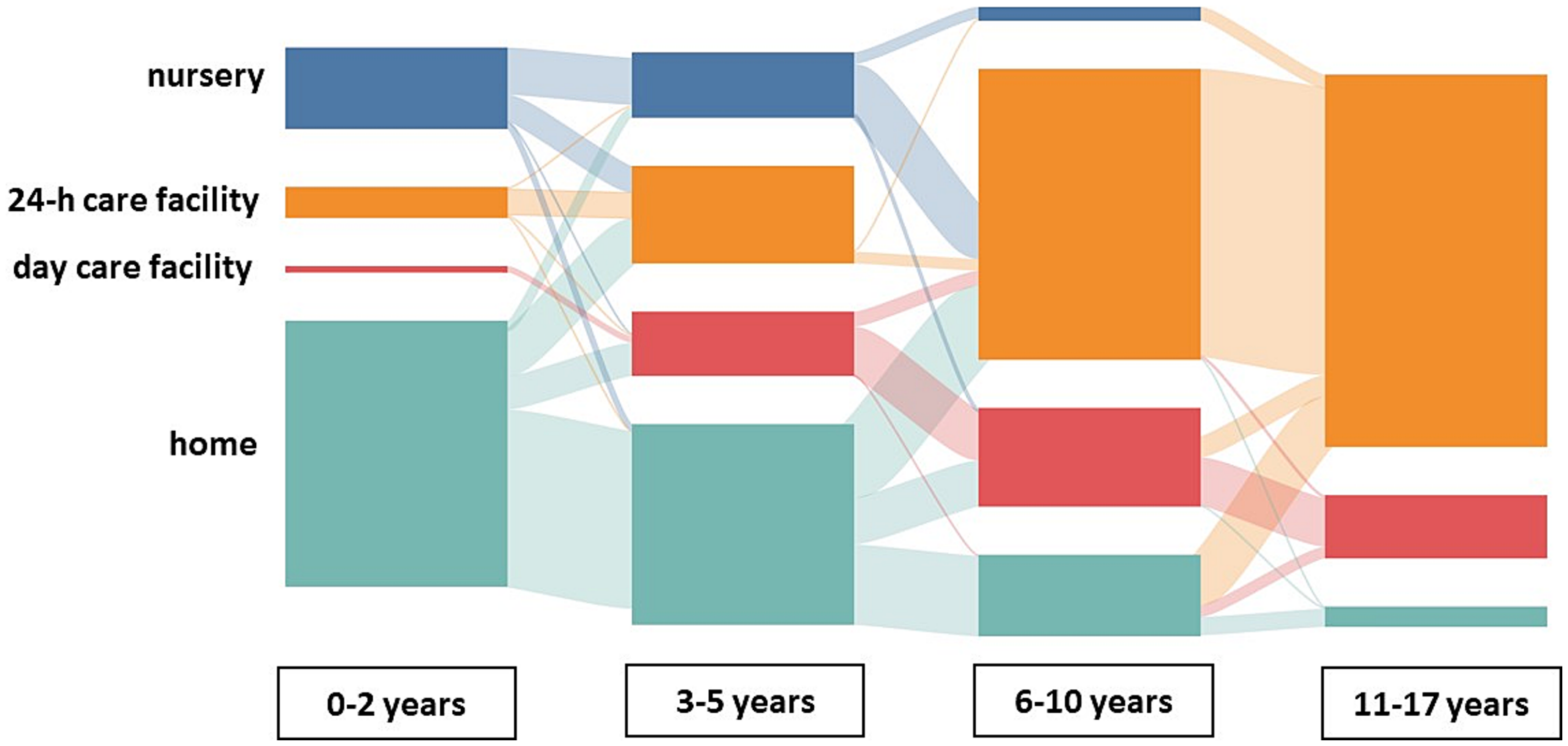
Care trajectories between birth and 18 years old *N* = 407.

Figure 1 illustrates the care trajectories (Sankey diagram) across the four predefined life periods, summarized by the primary care modalities during each period, for the 407 individuals included in the analysis.

As shown in the diagram, a large proportion of the sample was cared for exclusively at home during the period from birth to 2 years of age. This proportion gradually decreases as individuals grow older and, during the period from 11 to 17 years of age, it is observed only in a very small minority of individuals. Flow analysis reveals that individuals initially cared for exclusively at home are more likely to transition to 24-h care facilities than to day care facilities.

In contrast, 24-h care facilities show an opposite trend, with their use increasing progressively as individuals grow older. During the period from 11 to 17 years of age, 24-h care facilities represent the predominant care modality. Day care facilities never represent the dominant care modality at any period of life. At every transition point, part of this group shifts to 24-h care facilities.

Most children who were in nursery during the first period studied (birth to 2 years) remained in nursery during the second period (3 to 5 years), while a smaller portion moved to 24-h care facilities. Unexpectedly, a small proportion continued to be cared for in nursery during the third period, 6 to 10 years.

The Supplementary file 1 provides the care trajectories across the four predefined life periods for the subsample of individuals aged 18 to 35 years. The findings are consistent with those reported for the entire sample. Details are provided in Supplementary file 2.

### 3.3 Time spent in the primary care modality according to the life periods

**Figure 2:**
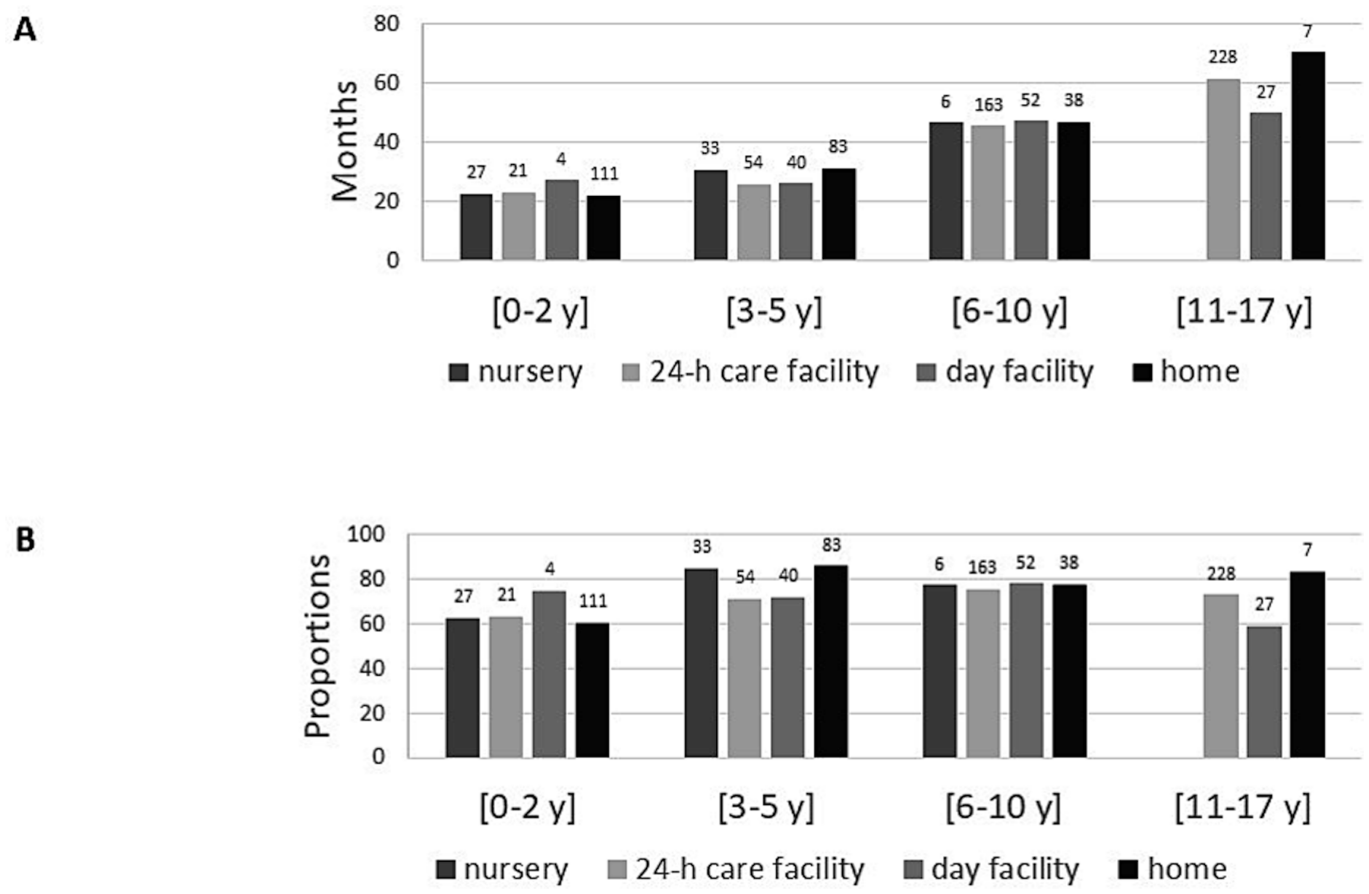
Time spent in primary care modality across life periods. **(A)**. Means of time spent in the primary care modality according to the four life periods. **(B)**. Proportions of time spent in the primary care modality according to the four life periods.

The time spent in the primary care modality during the 0–2-year life period ranged on average between 22 and 27 months, depending on the type of care setting, which corresponds to approximately 60 to 75% of the total duration of this period. For the 3–5-year period, which is of the same duration as the previous one, the average time spent in the primary care setting was longer, ranging from 25 to 31 months, corresponding to 71 to 86% of the total duration of that period. For the 6–10-year life period, which is longer (5 years), the time spent in the primary care setting was also longer, averaging between 45 and 47 months, or approximately 76% of the total period. These values were relatively consistent across care modalities. For the last and longest period (11–17 years old), the results are less homogeneous and vary depending on the care modality. The findings are illustrated in Figure 2 (A: means; B: proportions).

### 3.4 Care discontinuity

**Figure 3:**
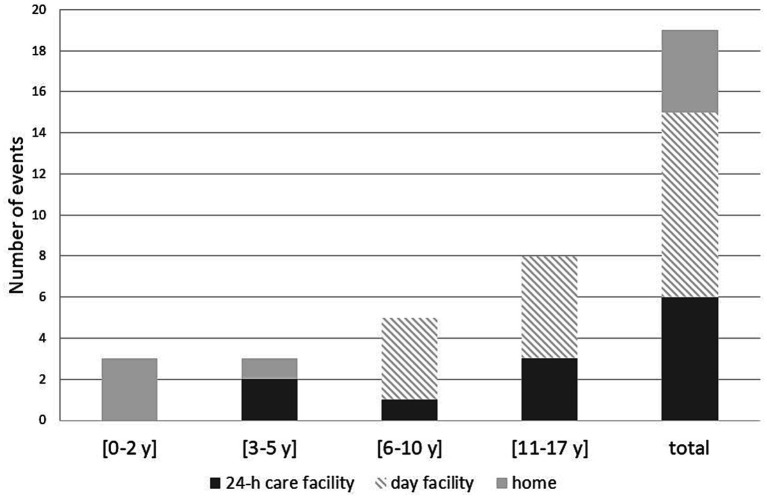
Care discontinuity according to the four life periods.

A total of 19 care discontinuity events—representing less than 5% of the sample—were reported, occurring across the following three care modalities: home care, day care facilities, and 24-h care facilities. The Figure 3 illustrates these findings.

## 4 Discussion

To our knowledge, this is the first study conducted in France focusing specifically on the care trajectories of persons with polyhandicap. Based on a large sample of individuals under 35 years old, this study describes movements between care modalities across four predefined life periods from birth to 18 years. This work provides a first empirical basis to document and identify transition patterns and potential points of rupture within these care pathways.

The first key observation is the high proportion of individuals exclusively cared for at home during the first life period, from birth to 2 years. As they advance in age, these individuals progressively move into other types of care modalities. Some children shift to day care structures, which allow them to spend time outside the home and take advantage of the educational supports provided by the structures. This option is also probably beneficial for families, as it offers them respite and time not solely focused on caregiving. However, it is noteworthy that the flow most often occurs directly towards a 24-h care facility, without an intermediate step in a day care facility. Two hypotheses can be proposed: either these families did not have access to day care facilities, or they had been waiting for a place in a 24-h care facility for a long time.

The second finding concerns the increasing importance of 24-h care facilities as the child grows, until this becomes the most frequent care modality during the 11–17 years life period. Several explanations may be proposed to account for this trend: families cannot find available places in day care facilities, or families are no longer able to provide care themselves and thus seek full-time care services. It is important to distinguish between these situations because the appropriate responses to each differ. In the first case, efforts should focus on increasing the availability of day care facility places; in the second, improving support for families—both financial and organizational—is essential. In some contexts, particularly those promoting deinstitutionalization, this trend may differ in magnitude and timing depending on national care systems and the availability of community-based services (2). Our data do not allow us to disentangle the respective contributions of clinical deterioration and system-level constraints, and both are likely to play a role. Using qualitative methods could yield a better comprehension of these mechanisms, particularly how families make decisions, experience care constraints, and navigate available services.

In this sample, day care facilities are not the predominant form of care. There is a noticeable increase during the early stages of life, but between the ages of 11 and 17, this trend reverses in favor of 24-h care facilities. Several explanations are possible: either the number of available facilities is insufficient, or they are perceived as inappropriate, or families may prefer a more comprehensive form of care. There is an urgent need for policymakers to examine these issues to develop and implement suitable actions for improvement. In the current literature, very few studies provide detailed quantitative descriptions of how time is distributed between day care and 24-h care facilities in children with PIMD/polyhandicap, either in France or in other countries. As a result, our findings should mainly be interpreted within the French context, where community-based alternatives remain limited, and may not be directly generalizable to health systems that have more advanced deinstitutionalization policies (2, 14).

In addition, these trajectories should be interpreted in light of the clinical profiles of the children and the organization of the French care system. In theory, different types of institutions correspond to different levels of clinical severity: rehabilitation facilities (SMR) are expected to accommodate children with more complex medical needs, requiring intensive healthcare, whereas medico-social institutions are designed for children with less medical severity and a greater need for educational support. However, previous studies have shown that this theoretical alignment is only partially achieved in practice (5, 15), with some children with severe conditions being cared for in medico-social settings, and conversely. Several hypotheses may explain this mismatch. Structural constraints—such as limited availability of places or geographical distance from the family home—may lead to care orientations that do not fully correspond to the child’s clinical needs. In addition, family preferences and capacities, as well as broader contextual factors such as regional disparities or socioeconomic conditions, may also influence these trajectories. These interpretations should however be considered with caution, as our data do not allow us to formally disentangle the respective contributions of clinical needs and system-level factors. As a result, the observed transitions likely reflect a combination of clinical needs and healthcare system constraints, rather than purely medically driven trajectories.

While the average duration spent in care settings appears long, these results should be interpreted with caution, as they reflect only the child’s situation in their primary care modality. It may mask more complex or unstable care trajectories as described by some families who spend several years seeking out the care arrangement they feel is most appropriate for their child. In all the life periods studied, time spent in the primary care modality rarely surpasses 80%, indicating that most children experienced at least one additional care setting during each age period.

Lastly, the number of care discontinuity events may appear low. However, each of these events still represents a real challenge for the families who experience them. All types of care modalities appear to be affected. Once again, a more in-depth understanding of the underlying mechanisms is needed in order to improve the continuity and coordination of care.

## 5 Limitations

The representativeness of the sample should be considered with caution, as the cohort was not designed to provide nationally representative estimates but rather to describe care trajectories within a large, multicenter sample. However, we can reasonably assume a good level of representativeness for individuals cared for in SMR settings, as a substantial proportion of national capacity is covered by the participating centers. In contrast, the representativeness of medico-social facilities is more uncertain, as their inclusion was based on voluntary participation.

Although the sample size of this study is relatively large compared to what is available in the literature, it is essential to confirm these findings in larger and more representative populations. For instance, individuals receiving exclusively home-based care are underrepresented in the EVAL-PLH cohort, whose inclusion criteria are mainly based on institutional settings. The third wave of the EVAL-PLH cohort, scheduled between 2025 and 2026, will help partially address this limitation by specifically expanding the inclusion of this population. In addition, analyses based on large national databases—such as the National Health Data System (in French: Système National des Données de Santé)—will provide further robust insights into these issues (16, 17). Furthermore, this work lays the groundwork for future analyses using modeling methods, such as clustering or sequence analysis, aimed at correlating these trajectories with patients’ clinical profiles (e.g., GMFCS levels, medical stability, or intellectual impairment). However, such approaches require larger sample sizes to ensure the stability and interpretability of the identified patterns; given the current sample size, attempting these analyses would have entailed a substantial risk of overfitting and unstable cluster solutions.

The analysis considered only the primary care modality for each life period, which simplifies the complexity of actual care situations. Future research will need to explore more detailed levels of granularity to gain a deeper understanding of individual care trajectories.

This study did not examine the reasons underlying changes—or lack of changes—in care trajectories. These factors are essential to understanding the barriers involved, which may be organizational, personal, or related to reluctance and individual beliefs (18). Further research, likely involving qualitative or mixed-methods approaches, will be needed to investigate these aspects more thoroughly.

Finally, the findings should be interpreted in light of the French healthcare context, as this work is intended to provide a detailed analysis of the national organization of institutional care. However, these results may offer useful insights for other healthcare systems with similar structures or facing comparable challenges.

## 6 Conclusion

Knowledge about the care pathways of individuals with PIMD/polyhandicap remains fragmented. This study provides initial findings by describing the life trajectories of these individuals from birth to the age of 18, covering a continuum from home-only care to placement in 24-h care facilities. This understanding is essential to support policymakers in developing appropriate actions and evidence-based improvements to care provision.

## Data Availability

Authors are not able to share the data set publicly available for legal or ethical reasons. The data include potentially identifying and sensitive person information. The data are only available upon request from the sponsor (Assistance Publique des Hôpitaux de Marseille, Direction de la Recherche en Santé, 80 rue Brochier, Marseille, France, aap.drs@ap-hm.fr or the study coordinator Karine Baumstarck, karine.baumstarck@univ-amu.fr).
