# Supplementary material for: Care trajectories in children with profound intellectual and multiple disabilities/polyhandicap: a cross-sectional study of the French National Cohort
Source: Front Public Health. 2026 Jul 3;14:1873749. doi: 10.3389/fpubh.2026.1873749 (PMC13375974; doi:10.3389/fpubh.2026.1873749)
Supplement: Supplementary file 1 [file Data_Sheet_1.docx]

**Additional file 1. Care trajectories between birth and 18 years old: people aged from 18-35 years N=205**


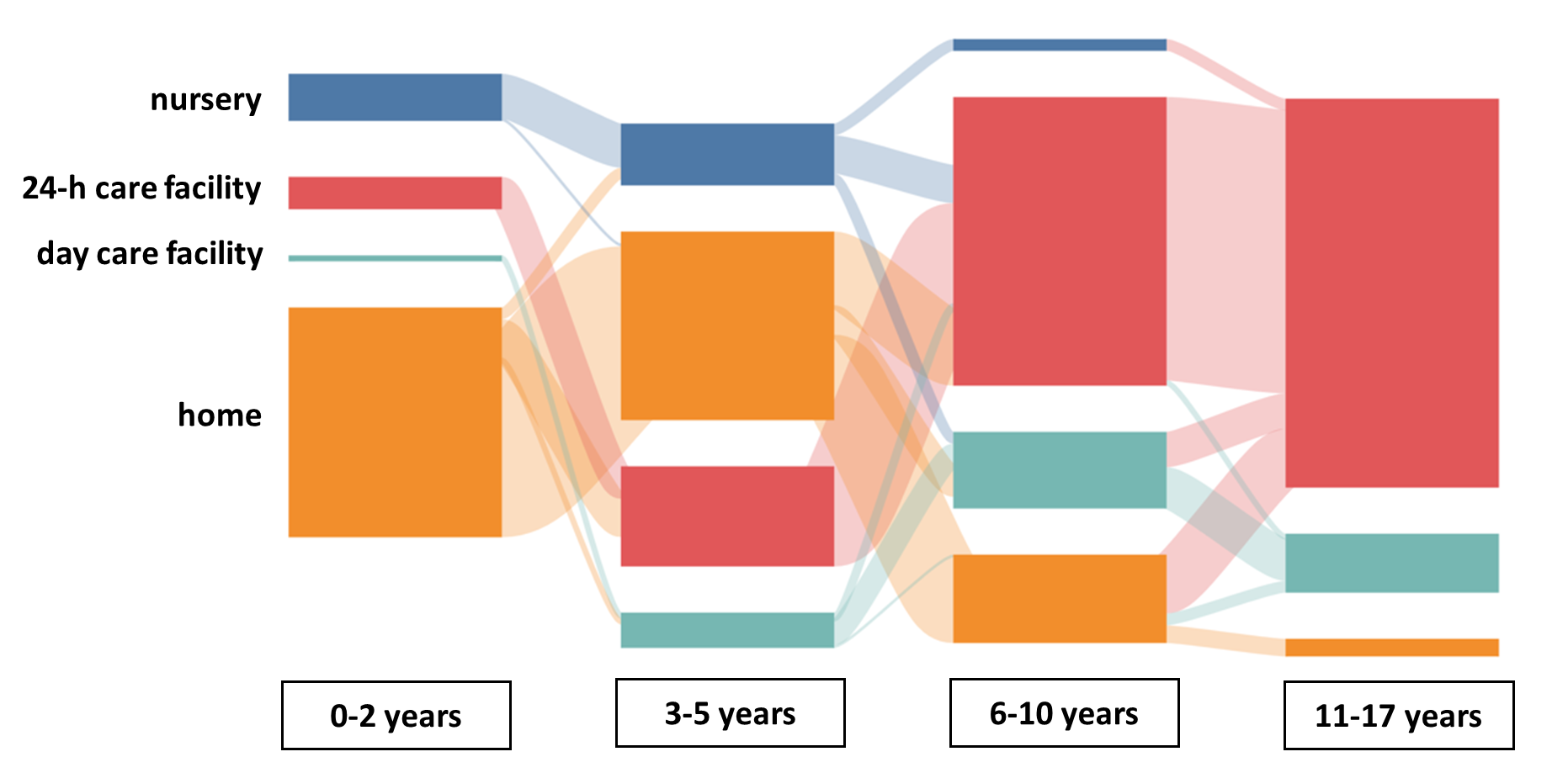


**Additional file 2. Origins, targets, and flow values for the whole sample and for the subsample of people aged from 18 to 35 years**

| **The whole sample N=407** | | | | | | | | | | |
| --- | --- | --- | --- | --- | --- | --- | --- | --- | --- | --- |
| **Origin** |  | **Target** |  | **Origin** |  | **Target** |  | **Origin** |  | **Target** |
| **[0-2 y]** | **N** | **[3-5 y]** |  | **[3-5 y]** | **N** | **[6-10 y]** |  | **[6-10 y]** | **N** | **[11-17 y]** |
| nursery | 44 | nursery |  | nursery | 11 | nursery |  | nursery | 13 | 24-h care facility |
| nursery | 25 | 24-h care facility |  | nursery | 46 | 24-h care facility |  | 24-h care facility | 267 | 24-h care facility |
| nursery | 1 | day facility |  | nursery | 4 | day facility |  | 24-h care facility | 3 | day facility |
| nursery | 6 | home |  | 24-h care facility | 2 | nursery |  | 24-h care facility | 1 | home |
| 24-h care facility | 2 | nursery |  | 24-h care facility | 11 | 24-h care facility |  | day facility | 20 | 24-h care facility |
| 24-h care facility | 24 | 24-h care facility |  | day facility | 14 | 24-h care facility |  | day facility | 45 | day facility |
| 24-h care facility | 2 | day facility |  | day facility | 45 | day facility |  | day facility | 1 | home |
| 24-h care facility | 1 | home |  | day facility | 1 | home |  | home | 47 | 24-h care facility |
| day facility | 6 | day facility |  | home | 69 | 24-h care facility |  | home | 11 | day facility |
| home | 10 | nursery |  | home | 43 | day facility |  | home | 17 | home |
| home | 42 | 24-h care facility |  | home | 75 | home |  |  |  |  |
| home | 31 | day facility |  |  |  |  |  |  |  |  |
| home | 165 | home |  |  |  |  |  |  |  |  |
| **People aged from 18 to 35 years N=205** | | | | | | | | | | |
| **[0-2 y]** | **N** | **[3-5 y]** |  | **[3-5 y]** | **N** | **[6-10 y]** |  | **[6-10 y]** | **N** | **[11-17 y]** |
| nursery | 15 | nursery |  | nursery | 4 | nursery |  | nursery | 4 | 24-h care facility |
| nursery | 1 | home |  | nursery | 13 | 24-h care facility |  | 24-h care facility | 96 | 24-h care facility |
| 24-h care facility | 11 | 24-h care facility |  | nursery | 4 | day facility |  | 24-h care facility | 2 | day facility |
| day facility | 2 | day facility |  | 24-h care facility | 34 | 24-h care facility |  | day facility | 12 | 24-h care facility |
| home | 4 | nursery |  | day facility | 3 | 24-h care facility |  | day facility | 14 | day facility |
| home | 13 | 24-h care facility |  | day facility | 8 | day facility |  | home | 20 | 24-h care facility |
| home | 2 | day facility |  | day facility | 1 | home |  | home | 4 | day facility |
| home | 59 | home |  | home | 25 | 24-h care facility |  | home | 6 | home |
|  |  |  |  | home | 10 | day facility |  |  |  |  |
|  |  |  |  | home | 29 | home |  |  |  |  |
